# Optimal cut-off of homeostasis model assessment of insulin resistance (HOMA-IR) for the diagnosis of metabolic syndrome: third national surveillance of risk factors of non-communicable diseases in Iran (SuRFNCD-2007)

**DOI:** 10.1186/1743-7075-7-26

**Published:** 2010-04-07

**Authors:** Alireza Esteghamati, Haleh Ashraf, Omid Khalilzadeh, Ali Zandieh, Manouchehr Nakhjavani, Armin Rashidi, Mehrdad Haghazali, Fereshteh Asgari

**Affiliations:** 1 Endocrinology and Metabolism Research Center (EMRC), Vali-Asr Hospital, School of Medicine, Tehran University of Medical Sciences, Tehran, Iran; 2 Department of Cardiology, Tehran Heart Center, Tehran University of Medical Sciences, Tehran, Iran; 3 Center for Disease Control, Ministry of Health and Medical Education, Tehran, Iran

## Abstract

**Aim:**

We have recently determined the optimal cut-off of the homeostatic model assessment of insulin resistance for the diagnosis of insulin resistance (IR) and metabolic syndrome (MetS) in non-diabetic residents of Tehran, the capital of Iran. The aim of the present study is to establish the optimal cut-off at the national level in the Iranian population with and without diabetes.

**Methods:**

Data of the third National Surveillance of Risk Factors of Non-Communicable Diseases, available for 3,071 adult Iranian individuals aging 25-64 years were analyzed. MetS was defined according to the Adult Treatment Panel III (ATPIII) and International Diabetes Federation (IDF) criteria. HOMA-IR cut-offs from the 50th to the 95th percentile were calculated and sensitivity, specificity, and positive likelihood ratio for MetS diagnosis were determined. The receiver operating characteristic (ROC) curves of HOMA-IR for MetS diagnosis were depicted, and the optimal cut-offs were determined by two different methods: Youden index, and the shortest distance from the top left corner of the curve.

**Results:**

The area under the curve (AUC) (95%CI) was 0.650 (0.631-0.670) for IDF-defined MetS and 0.683 (0.664-0.703) with the ATPIII definition. The optimal HOMA-IR cut-off for the diagnosis of IDF- and ATPIII-defined MetS in non-diabetic individuals was 1.775 (sensitivity: 57.3%, specificity: 65.3%, with ATPIII; sensitivity: 55.9%, specificity: 64.7%, with IDF). The optimal cut-offs in diabetic individuals were 3.875 (sensitivity: 49.7%, specificity: 69.6%) and 4.325 (sensitivity: 45.4%, specificity: 69.0%) for ATPIII- and IDF-defined MetS, respectively.

**Conclusion:**

We determined the optimal HOMA-IR cut-off points for the diagnosis of MetS in the Iranian population with and without diabetes.

## Introduction

Insulin resistance, which represents a reduced physiological response of the peripheral tissues to the action of the normal levels of insulin, is amajor finding in several metabolic disorders, including type 2 diabetes and metabolic syndrome (MetS) [1]. Therefore, a reliable measure of insulin resistance is important for investigating the link between insulin resistance and MetS. Furthermore, given that insulin resistance is an important risk factor for development of type 2 diabetes and incident cardiovascular diseases, identification of subjects with insulin resistance is a strategy for identifying high-risk people for targeted preventive interventions [2,3].

The homeostasis model assessment of insulin resistance (HOMA-IR), which is developed for application in large epidemiologic investigations [4], is an alternative to the glucose clamp and the most commonly used surrogate measure of insulin resistance in vivo. In terms of precision (reproducibility of measure), HOMA-IR is comparable to the glucose clamp technique. HOMA-IR is inferior to the clamp technique in terms of accuracy, but using HOMA-IR makes it possible to study a large number of subjects and with a single glucose and insulin measurement in the fasting state [5]. Although the HOMA-IR has been widely used, its cut-off for insulin resistance has not been conclusive. In addition, the HOMA-IR cut-off points to diagnose insulin resistance cannot be readily applied to all populations and may vary from race to race [6-18]. In a recent study on 1,327 non-diabetic, normotensive individuals in Tehran, we demonstrated this cut-off to be 1.8 [10]. HOMA-IR may also serve as a surrogate measure of the insulin resistance phenotype, as it identifies a proportion of subjects with insulin resistance without directly measuring insulin action [19,20].

Population-based studies for defining cut-off values of insulin resistance for diagnosis of MetS are limited. In this study, we sought, for the first time, to evaluate the distribution and optimal cut-off value of HOMA-IR for identifying MetS in a Middle Eastern population with and without diabetes.

## Methods

### Subjects

The data obtained from the third National Surveillance of Risk Factors of Non-Communicable Diseases in Iran (SuRFNCD-2007) [21] were analyzed. SuRFNCDs are a series of health surveys designed based on the STEPwise guidelines of the WHO [22] to be representative of the Iranian adult population. The first, second and the third surveys were performed in 2005, 2006 and 2007, respectively. Further details can be found in our previous reports [21,23]. In this study, we used the data of blood pressure, waist circumference, height and weight as part of a standardized physical examination and data of diabetes and hypertension history as part of an interview. After excluding pregnant women and those with missing information on lipid profile, fasting glucose and insulin levels (n = 1,162), analysis was performed on a sample of 3,071 Iranians aged 25-64 years. The Institutional Review Board of Center for Disease Control (CDC) of Iran approved the study protocol, and all subjects gave verbal informed consent before participation.

### Clinical and laboratory data

Weight and height of participants were determined in light clothing and without shoes. Portable calibrated electronic weighing scale and portable measuring inflexible bars were used. Waist circumference was measured using constant tension tape at the end of a normal expiration, with arms relaxed at the sides, at the midpoint between the lower part of the lowest rib and the highest point of the hip on the mid-axillary line. Body mass index (BMI) was calculated as weight (in kilograms) divided by height (in meters) squared. Blood pressure was measured with a calibrated Omron M7 sphygmomanometer (HEM-780-E). The mean value of three measurements, made at intervals of 5 minutes, was used for analysis. Blood samples were collected following 12 h overnight fast. Fasting plasma glucose was measured by the enzymatic colorimetric method using glucose oxidize test (intra- and inter-assay coefficients of variation 2.1% and 2.6%, respectively). Serum total cholesterol, triglyceride, and high density lipoprotein-cholesterol (HDL-cholesterol) were determined by enzymatic methods (Parsazmun, Karaj, Iran). Low density lipoprotein-cholesterol (LDL-cholesterol) was calculated using the formula of Friedewald et al. [24]. When serum triglyceride concentration was greater than 400 mg/dl, LDL-cholesterol was determined directly by enzymatic method using commercial kits (Parsazmun, Karaj, Iran). Insulin was measured by radioimmunoassay (Immunotech, Prague, Czech Republic). Sensitivity was 0.5 μU/mL, and the upper limits of intra- and inter-assay coefficients of variation were 4.3 and 3.4, respectively. HOMA-IR was calculated as fasting insulin (U/l) × fasting glucose (mg/dl)/405, as described by Matthews et al. [4].

### Definition of MetS

MetS was defined according to the Adult Treatment Panel III (ATPIII) [25] and International Diabetes Federation (IDF) [26] criteria. Under the ATPIII criteria, MetS was defined as the presence of three or more of the following risk factors: abdominal obesity (waist circumference ≥102 cm [men] or ≥88 cm [women]), triglyceride ≥150 mg/dL, HDL-cholesterol <40 mg/dL (men) or <50 mg/dL (women), blood pressure ≥130/85 mmHg, and fasting plasma glucose ≥100 mg/dL (or diabetes) [27]. According to IDF definition, a person defined as having MetS must have central obesity (waist circumference >90 cm in males and females, based on cut-off points of the Iranian population [28]) plus any two of the following: 1) Triglyceride ≥150 mg/dL; 2) HDL-cholesterol <40 mg/dL for men, <50 mg/dL for women; 3) systolic blood pressure ≥130 mmHg or diastolic blood pressure ≥85 mmHg; 4) fasting plasma glucose ≥100 mg/dL (or diabetes) [26]. For both ATPIII and IDF definitions, subjects who were taking antihypertensive medication were considered hypertensive individuals. Those with triglyceride <150 mg/dL, HDL-cholesterol ≥40 mg/dL for men or ≥50 mg/dL for women, fasting plasma glucose <100 mg/dL, systolic blood pressure <130 mmHg, diastolic blood pressure <85 mmHg, serum total cholesterol ≤200 mg/dL, and BMI ≤25 kg/m^2 ^were defined as normal subjects (without any metabolic abnormality).

### Statistical analysis

Data were analyzed using the statistical software for social sciences (SPSS, Version 16 for Windows; SPSS Inc., Chicago, IL, USA). Data were directly weighted for age (10-year strata) and sex distribution of the Iranian population according to the results of the national census of Iran in 2006. Complex sample survey analysis was performed to standardize the results for the population of Iran. Continuous variables are expressed as mean ± standard error of the mean (SEM). HOMA-IR cut-offs from the 50th to the 95th percentile along with their corresponding sensitivity and specificity for diagnosis of IDF-defined MetS in non-diabetic, normal and diabetic individuals were calculated. The receiver operating characteristic (ROC) curve of HOMA-IR for the diagnosis of ATPIII- and IDF-defined MetS was depicted and the area under the curve (AUC) was calculated for diabetic and non-diabetic subjects separately. ROC curves are interpreted as the probability that the modeled phenotype(s) can correctly discriminate subjects developing end points from those without end points, where 0.5 is chance discrimination and 1.0 is perfect discrimination. To determine the optimal thresholds, the point on the ROC curve with maximum Youden index [sensitivity- (1-specificity)]), and the point with shortest distance value form the point (0,1) [(1 - sensitivity)^2 ^+ (1 - specificity)^2^] were calculated [29]. These are the two most commonly used methods for establishing the optimal cut-off [30]. We also calculated the positive likelihood ratio (PLR), which summarizes how likely patients with the disease are to have a specified test result compared with patients without the disease. PLR is calculated as sensitivity/(100%-specificity).

Primary analyses were performed without covariate adjustment to reflect standard use of blood test results in clinical practice. Subsidiary analyses of surrogate measures considered additional adjustment for age and sex. To control whether this would lead to over-fitting HOMA-IR in statistical models, analyses were repeated with fasting insulin as an alternative to HOMA-IR. For fasting insulin, we also considered additional adjustment for fasting glucose (to assess adjusted discrimination compared with discrimination using HOMA-IR). For each surrogate measure, we compared the ROC of the fuller model with that of the sparser model. P < 0.05 was considered statistically significant.

## Results

Table S1; Additional file 1 shows clinical and laboratory characteristics of the study participants. Fasting plasma insulin and glucose and as a result HOMA-IR were similar in both genders. Although a higher proportion of women had ATPIII-defined MetS (39.0% vs. 28.3% in men, P < 0.001), IDF-defined MetS prevalence was similar in both genders (34.0% in men and 35.5% in women).

Age and sex distribution of HOMA-IR cut-offs from the 50th to the 95th percentile along with their corresponding sensitivity and specificity for the diagnosis of IDF-MetS in non-diabetic and diabetic individuals are shown in Tables S2 and S3 (additional file 1), respectively. Regardless of the diabetes status, the prevalence of MetS was substantially higher in older ages for any given HOMA-IR threshold. Within diabetic and non-diabetic subjects, about half of participants with MetS had HOMA-IR levels in the upper 35% and 40% of the population distribution, respectively. The values of sensitivity and specificity that might be considered "acceptable" may differ depending on the clinical situation; Tables S2 and S3; Additional file 1 can be used to assess various combinations. For instance, assume that > 80% sensitivity represents acceptable test performance; the HOMA-IR threshold associated with > 80% sensitivity for IDF-defined MetS was 1.35 in non-diabetics (specificity: 33%) and 2.2 in diabetics (specificity: 45.5%), which corresponds to the 32nd percentile or greater for non-diabetics and the 22nd percentile or greater for diabetics, respectively. In non-diabetic and diabetic individuals, the HOMA-IR threshold that yielded >80% specificity was 75th percentile (i.e. 2.20 in non-diabetics and 5.8 in diabetics). This cut-off yielded lower sensitivity in diabetic individuals (29% vs. 35% in non-diabetics).

**Figure 1:**
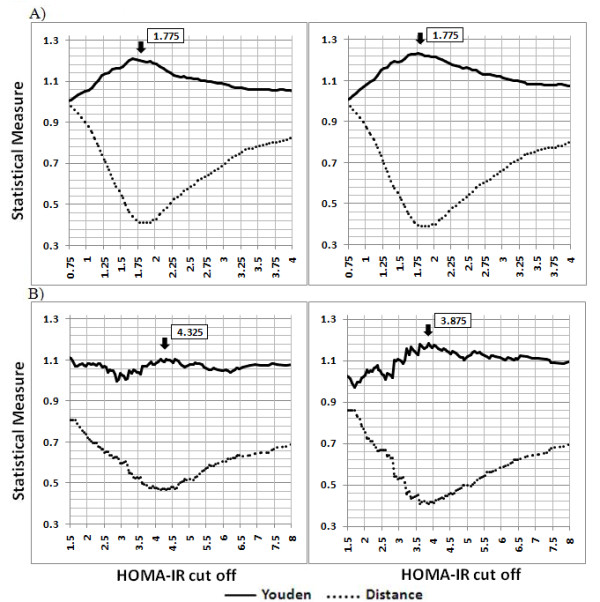
**The optimal cut point of homeostasis model assessment (HOMA) for diagnosis of metabolic syndrome**. The diagnostic criteria for metabolic syndrome are those recommended by the international diabetes federation (IDF) (left) and Adult Treatment Panel III (ATP III) (right). The top panels (A) show the results in non-diabetic individuals and the bottom panels (B) refer to diabetic individuals.

Youden index values and the distance from the top left corner of the ROC curve of HOMA-IR for diagnosis of IDF- and ATPIII-defined MetS are depicted in Figure 1. In non-diabetic individuals, HOMA-IR ranged from 1.75 to 2 (corresponding to the 57th to the 68th percentile), show a plateau on the top of Youden index curve, and at the bottom of distance curve. The cut-off 1.775 is the best threshold for MetS diagnosis by both definitions; it maximized Youden index and minimized the distance on the ROC curve (ATPIII: sensitivity = 57.3%, specificity = 65.3%, Youden index = 1.230, distance = 0.394; IDF: sensitivity = 55.9%, specificity = 64.7%, Youden index = 1.202, distance = 0.413). Using this cut off, the prevalence of insulin resistance among those with and without ATPIII-defined MetS was 44.0% and 23.8% (p < 0.0001) respectively. For those with and without IDF defined-MetS, the prevalence rates were 42.5% and 24.1% (p < 0.0001), respectively. Approximately 33.6% of subjects who met neither ATPIII- nor IDF-MetS definitions were insulin resistant.

In diabetic individuals, we observed a plateau in HOMA-IR values around 4. The optimal cut-off of HOMA-IR for MetS diagnosis in this group was 3.875 (sensitivity = 49.7%, specificity = 69.6%, Youden index = 1.118, distance = 0.410) for ATPIII-defined MetS and 4.325 for IDF-defined MetS (sensitivity = 45.4%, specificity = 69.0% Youden index = 1.105, distance = 0.467).

**Figure 2:**
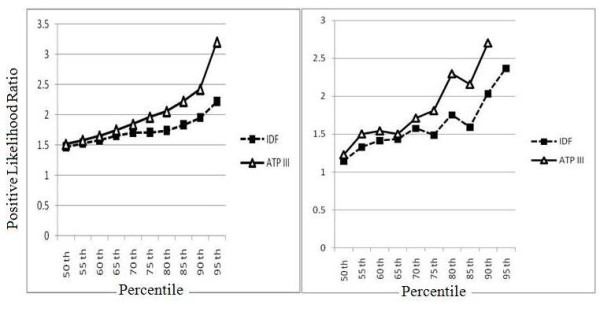
**Positive likelihood ratios of different HOMA-IR percentiles for prediction of IDF- and ATPIII-defined metabolic syndrome**. The results in non-diabetic and diabetic individuals are shown in the left and right panels, respectively.

As depicted in Figure 2, the likelihood of MetS increased steadily with increasing percentiles of HOMA-IR, with a threshold at the 90th percentile in non-diabetics and 85th percentile in diabetics. Likelihood ratios for ATPIII were higher than for IDF, especially at higher percentiles of HOMA-IR. HOMA-IR and fasting insulin levels were significantly correlated (r = 0.46, P < 0.0001). HOMA-IR significantly increased with rising numbers of MetS components (p < 0.001).

ROC analyses showed that the diagnosis of MetS is made more accurately by using HOMA-IR than fasting insulin. Insulin resistance (using any surrogate) or fasting insulin predicts ATPIII-defined MetS more accurately than IDF-defined MetS. For example, the HOMA-IR AUC (95% CI) for IDF-defined MetS was 0.650 (0.631-0.670) compared with 0.683 (0.664-0.703) for ATPIII-defined MetS. One reason that fasting insulin underperformed HOMA-IR is that information about plasma glucose is contained within the latter measure. Additional adjustment for age and sex was performed in subsidiary analyses. This did not influence the accuracy of MetS diagnosis by both definitions, i.e. crude versus age- and sex-adjusted ROCs (for both HOMA-IR and fasting insulin models) had statistically equal performance. Adjustment for fasting glucose decreased the accuracy of MetS diagnosis by both HOMA-IR and fasting insulin (data not shown).

## Discussion

We demonstrated that the risk of MetS increased with rising HOMA-IR percentiles. The optimal cut-off point of HOMA-IR for the diagnosis of MetS in our population was estimated to be 1.775 in non-diabetics and around 4 in diabetic patients. In line with previous population-based studies [20,31], we found that insulin resistance and MetS were significantly associated, and HOMA-IR levels were directly related to the number of MetS components. The prevalence of insulin resistance was notably higher among those who met either ATPIII or IDF criteria of MetS, and with increasing HOMA-IR percentiles the risk gradients were greater for ATPIII- than for IDF-defined MetS (Figure 2). Similarly, insulin resistance predicted incident ATPIII-MetS more accurately than IDF-MetS. Nevertheless, MetS definitions did not provide a sensitive approach to identify insulin-resistant individuals and approximately one third of subjects who met neither ATPIII nor IDF definitions of MetS were insulin resistant.

HOMA-IR, developed in 1985 by Matthews and co-workers [4], was used in this study as it is a simple and appropriate method in epidemiological studies where dynamic studies like the euglycaemic glucose clamp technique, though the gold standard, may not be feasible due to the degree of sophistication and cost of necessary equipments [32]. The HOMA-IR method requires measuring a single fasting plasma glucose and the corresponding fasting plasma insulin level [4]. A current uncertainty is the clinical value of HOMA-IR or any surrogate insulin resistance measure for use in management or clinical prediction of metabolic disorders. The major shortcoming of the method is that the model applies values generated from lean young adults (less than 35 years old) of Caucasian origin as standard to other subjects [4,33]. Values for older adults would probably be different from those documented for this younger group, as older individuals are known to be relatively more insulin resistant [34]. Furthermore, ethnic and racial factors are known to be significant in the etiology of insulin resistance [35]. As a result of such factors, one important point in implementing the HOMA-IR method successfully is the presence of specific cut-points for the race or age of the studied population.

For inter-population comparisons, it is necessary to know normal values of HOMA-IR for each population. Although HOMA-IR has been widely used, there is hardly any consensus on the cut-off points for classification of insulin resistance. Some authors have tried to find HOMA-IR cut-offs in subjects who had increased tendencies toward insulin resistance or MetS, but their findings were not consistent [6-18]. Table S4; additional file 1, summarizes the available reports. Some of the inconsistencies may be due to the different clinical settings and ethnicity. Also, there is not a worldwide standardized assay for insulin. Different assays may produce different results for HOMA-IR [36]. Using different criteria to define insulin resistance and different approaches to determine cut-off values are other reasons for inconsistencies among studies. Some authors have used ROC curves for cut-off estimation [10,11,15,18]. Youden index and the distance from the top left corner of the ROC curve are two methods commonly used in previous work to determine the best HOMA-IR cut-off. Values based on median [7,8,12,16], 75th percentile [6], 90th percentile [9,14,17], lower boundary of the top quintile [10,13] or tertile [37] of HOMA-IR obtained from population studies or non-obese subjects with no metabolic disorders have been used previously.

Different cut-off points might be selected to optimize sensitivity versus specificity depending on the purpose. A screening test requires high sensitivity and moderate specificity, whereas a diagnostic test requires a much higher specificity. Although insulin resistance may be at the core of the cluster of metabolic abnormalities that characterizes MetS, our data suggest that MetS, defined by conventional criteria, is not always synonymous with insulin resistance [17,38]. The relationship between MetS and insulin resistance in the present study was not as strong as suggested by previous reports [17,38]. Although insulin resistance is the basic defect leading to MetS [27], neither insulin resistance nor hyperinsulinemia were among ATPIII or IDF criteria. Only the European Group for the Study of Insulin Resistance definition [39] requires the presence of insulin resistance to define "insulin resistance syndrome". The decision of the ATPIII or IDF to use putative manifestations of insulin resistance and compensatory hyperinsulinemia to diagnose MetS is based on the fact that specific measurements of insulin resistance are not clinically practical to predict insulin resistance [12].

The prevalence of MetS in our sample was 33.6% and 34.8% for ATPIII and IDF definitions, respectively. Our results regarding MetS prevalence, insulin levels, and HOMA-IR values suggest that women have a higher propensity to insulin resistance. The available reports on the prevalence of MetS show variable results (23%-40%), depending on ethnicity and the criteria used [40]. In addition to the role of genetic factors in predisposition to MetS [41], the high prevalence of the syndrome in our population is, at least in part, attributed to dramatic lifestyle changes during the past decade. Given that insulin resistance is an early step in the pathogenesis of type 2 diabetes [1], the high prevalence of insulin resistance in Iran, especially among the young, predicts an increasing burden of type 2 diabetes in the near future.

In conclusion, we showed that risk for MetS increases with increasing HOMA-IR percentiles. The optimal cut-off point of HOMA-IR for MetS diagnosis is 1.775 in non-diabetics and approximately 4 in diabetic individuals. Further prospective studies are warranted to elucidate the performance of these cut-offs in predicting incident diabetes or cerebrovascular disease in our country. A fairly large proportion of our participants were excluded because of missing lab results. Although excluded participants were randomly scattered across age, sex, BMI, and residential area categories of SuRFNCD-2007, and their exclusion is thus unlikely to have caused a significant problem in our analysis, this can be considered as a limitation of our study and is to be addressed in future work.

## Conflict of interests

The authors declare that they have no competing interests.

## Authors' contributions

AE participated in the design of the study and interpreted the results. HA and OK participated in statistical analysis, interpreted the results, and wrote the manuscript. AZ helped with statistical analysis and writing the manuscript. MN and AR interpreted the results and wrote the manuscript. MH and FA participated in the design of the study and conducting it. All authors read and approved the final manuscript.

## Supplementary Material

Additional file 1 **Supplementary Tables 1-4**. Table S1 - Clinical and laboratory characteristics of participants, Table S2 - Age and sex distribution of HOMA-IR values in non-diabetic subjects (n = 2,705). Table S3 - Age and sex distribution of HOMA-IR values in diabetic subjects (n = 366). Table S4 - Summary of reports (sorted by sample size) on HOMA-IR cut-off in different populations. Click here for file
