# Supplementary material for: Optimal cut-off of homeostasis model assessment of insulin resistance (HOMA-IR) for the diagnosis of metabolic syndrome: third national surveillance of risk factors of non-communicable diseases in Iran (SuRFNCD-2007)
Source: Nutr Metab (Lond). 2010 Apr 7;7:26. doi: 10.1186/1743-7075-7-26 (PMC2857836; doi:10.1186/1743-7075-7-26)
Supplement: Additional file 1 — Supplementary Tables 1-4. Table S1 - Clinical and laboratory characteristics of participants, Table S2 - Age and sex distribution of HOMA-IR values in non-diabetic subjects (n = 2,705). Table S3 - Age and sex distribution of HOMA-IR values in diabetic subjects (n = 366). Table S4 - Summary of reports (sorted by sample size) on HOMA-IR cut-off in different populations. [file 1743-7075-7-26-S1.DOC]

**Table S1. Clinical and laboratory characteristics of participants**

|  | Women (n = 1,590) | Men (n = 1,481) | Total (n = 3,071) |
| --- | --- | --- | --- |
| Waist Circumference(cm) | 88.8 ± 0.6 | 88.8 ± 0.5 | 88.8 ± 0.4 |
| BMI(kg/m^2^)*** | 27.5 ± 0.2 | 25.4 ± 0.2 | 26.4 ± 0.1 |
| Systolic Blood Pressure(mmHg) ** | 121.3 ± 0.8 | 124.6 ± 0.6 | 123.0 ± 0.5 |
| Diastolic Blood Pressure(mmHg)* | 81.3 ± 0.5 | 79.9 ± 0. | 80.6 ± 0.3 |
| Triglyceride(mg/dl)*** | 138.6 ± 2.7 | 158.7 ± 3.6 | 148.8 ± 2.4 |
| Total Cholesterol(mg/dl)*** | 199.4 ± 1.3 | 191.2 ± 1.4 | 195.3 ± 1.0 |
| HDL-Cholesterol (mg/dl)*** | 39.4 ± 0.3 | 34.4 ± 0.4 | 36.9 ± 0.3 |
| LDL-Cholesterol (mg/dl)** | 165.0 ± 7.5 | 214.0 ± 16.0 | 189.9 ± 9.0 |
| Fasting Insulin (U/l) | 9.85 ± 0.2 | 9.27 ± 0.2 | 9.56 ± 0.1 |
| Fasting Plasma Glucose (mg/dL) | 89.3 ± 0.5 | 91.3 ± 1.1 | 90.3 ± 0.6 |
| HOMA-IR (units) | 2.30 ± 0.05 | 2.31 ± 0.1 | 2.31 ± 0.05 |
| Metabolic syndrome |  |  |  |
| ATPIII *** | 39.0 % | 28.3 % | 33.6 % |
| IDF | 34.0 % | 35.5 % | 34.8 % |

Data are presented as mean ± standard error of the mean, or prevalence rates

Data are weighted and standardized for age distribution of the population of Iran in 2006.

BMI, body mass index; HOMA-IR, homeostasis model assessment of insulin resistance; ATPIII, Third Adult Treatment Panel; IDF, International Diabetes Federation

***: p < 0.05, **: p < 0.01, ***: p < 0.001

**Table S2.** Age and sex distribution of HOMA-IR values in non-diabetic subjects (n = 2,705).

|  | | Percentiles of HOMA-IR | | | | | | | | | |
| --- | --- | --- | --- | --- | --- | --- | --- | --- | --- | --- | --- |
|  |  | 50^th^ | 55^th^ | 60^th^ | 65^th^ | 70^th^ | 75^th^ | 80^th^ | 85^th^ | 90^th^ | 95^th^ |
| **All** ^a^ |  |  |  |  |  |  |  |  |  |  |  |
|  | Cut-off | 1.64 | 1.71 | 1.82 | 1.93 | 2.05 | 2.20 | 2.42 | 2.73 | 3.24 | 4.20 |
|  | Sen%/spe% | 64.1/56.2 | 59.7/61.2 | 53.8/66.0 | 48.1/70.9 | 41.9/75.4 | 35.0/79.5 | 27.9/83.9 | 21.8/88.1 | 15.0/92.3 | 8.2/96.3 |
|  | MetS^1^ (%) | 40.6 | 41.8 | 42.5 | 43.6 | 44.3 | 44.4 | 44.7 | 46.2 | 47.6 | 51.0 |
| **Sex** ^b^ |  |  |  |  |  |  |  |  |  |  |  |
| Women | Cut-off | 1.70 | 1.80 | 1.90 | 2.01 | 2.12 | 2.30 | 2.52 | 2.85 | 3.36 | 4.37 |
|  | Sen%/spe% | 62.4/55.5 | 57.1/60.5 | 51.5/64.8 | 46.2/69.8 | 41.9/74.6 | 32.5/78.4 | 27.8/83.8 | 22.1/87.9 | 14.3/92.0 | 8.1/96.2 |
|  | MetS (%) | 38.9 | 39.2 | 39.5 | 40.5 | 42.4 | 40.1 | 43.4 | 44.8 | 44.3 | 49.1 |
| Men | Cut-off | 1.58 | 1.66 | 1.73 | 1.85 | 1.97 | 2.10 | 2.27 | 2.59 | 3.11 | 3.99 |
|  | Sen%/spe% | 64.4/56.7 | 60.9/62.8 | 55.8/68.0 | 50.4/72.3 | 44.1/76.0 | 36.4/80.6 | 29.5/84.3 | 21.9/88.2 | 15.6/92.2 | 8.2/96.5 |
|  | MetS (%) | 42.0 | 44.4 | 45.9 | 47.0 | 47.2 | 47.8 | 47.8 | 47.5 | 49.3 | 53.4 |
| **Age** ^c^ |  |  |  |  |  |  |  |  |  |  |  |
| 25-34 yr | Cut-off | 1.63 | 1.69 | 1.78 | 1.90 | 2.01 | 2.12 | 2.31 | 2.58 | 3.15 | 4.13 |
|  | Sen%/spe% | 65.6/53.0 | 61.2/58.8 | 57.7/63.7 | 53.4/68.2 | 44.9/73.4 | 40.8/77.9 | 28.1/81.9 | 23.0/86.9 | 16.4/91.3 | 8.7/95.9 |
|  | MetS (%) | 25.0 | 26.1 | 27.5 | 28.6 | 28.7 | 30.6 | 27.1 | 29.5 | 31.1 | 39.9 |
| 35-44 yr | Cut-off | 1.66 | 1.73 | 1.83 | 1.95 | 2.07 | 2.25 | 2.48 | 2.88 | 3.52 | 4.53 |
|  | Sen%/spe% | 61.4/56.9 | 56.9/61.0 | 50.5/66.5 | 46.2/69.2 | 39.0/75.4 | 33.1/79.0 | 26.5/83.9 | 21.6/89.1 | 15.1/93.4 | 8.4/96.9 |
|  | MetS (%) | 45.6 | 46.2 | 47.0 | 48.4 | 48.2 | 48.1 | 49.1 | 53.8 | 57.4 | 61.3 |
| 45-54 yr | Cut-off | 1.67 | 1.74 | 1.85 | 1.97 | 2.10 | 2.25 | 2.43 | 2.72 | 3.15 | 4.20 |
|  | Sen%/spe% | 67.6/64.0 | 61.3/67.6 | 53.4/70.6 | 48.4/75.6 | 41.1/78.8 | 34.2/81.6 | 27.6/85.6 | 21.8/89.8 | 14.9/93.8 | 8.1/96.6 |
|  | MetS (%) | 59.1 | 59.3 | 58.2 | 60.3 | 59.9 | 58.8 | 59.5 | 62.2 | 64.9 | 68.4 |
| 55-64 yr | Cut-off | 1.65 | 1.76 | 1.85 | 1.98 | 2.11 | 2.27 | 2.44 | 2.89 | 3.27 | 4.34 |
|  | Sen%/spe% | 59.1/58.4 | 54.8/65.3 | 50.9/70.9 | 44.1/74.6 | 39.1/78.7 | 31.9/81.7 | 27.2/87.1 | 19.0/89.4 | 12.2/92.1 | 6.1/95.9 |
|  | MetS (%) | 60.1 | 62.6 | 65.0 | 64.8 | 66.1 | 65.0 | 69.2 | 65.5 | 62.0 | 61.1 |

^a^ weighted and standardized for age and sex, ^b^ for age, and ^c^ for sex distribution of the population of Iran in 2006.

^1^: IDF definition

**Table S3.** Age and sex distribution of HOMA-IR values in diabetic subjects (n = 366).

|  | | Percentiles of HOMA-IR | | | | | | | | | |
| --- | --- | --- | --- | --- | --- | --- | --- | --- | --- | --- | --- |
|  |  | 50^th^ | 55^th^ | 60^th^ | 65^th^ | 70^th^ | 75^th^ | 80^th^ | 85^th^ | 90^th^ | 95^th^ |
| **All** |  |  |  |  |  |  |  |  |  |  |  |
|  | Cut | 3.53 | 3.80 | 4.32 | 4.77 | 5.27 | 5.80 | 6.95 | 8.63 | 12.94 | 18.01 |
|  | Sen%/spe% | 53.7/53.1 | 50.8/61.8 | 46.1/67.4 | 40.1/72.0 | 35.6/77.4 | 29.3/80.3 | 24.9/85.8 | 18.0/88.7 | 12.6/93.8 | 7.1/97.0 |
|  | MetS^1^ (%) | 65.6 | 69.0 | 70.2 | 70.6 | 72.4 | 71.2 | 74.5 | 72.7 | 77.2 | 79.6 |
| **Sex** |  |  |  |  |  |  |  |  |  |  |  |
| Women | Cut | 3.40 | 3.58 | 3.88 | 4.32 | 4.88 | 5.25 | 5.80 | 7.43 | 8.10 | 13.68 |
|  | Sen%/spe% | 50.0/46.3 | 47.1/47.1 | 45.0/65.2 | 39.7/67.5 | 30.2/69.4 | 25.9/75.2 | 21.8/80.4 | 16.0/88.0 | 10.8/91.6 | 5.3/94.3 |
|  | MetS (%) | 59.9 | 58.9 | 67.5 | 66.2 | 61.4 | 62.7 | 64.2 | 68.2 | 67.3 | 59.8 |
| Men | Cut | 3.68 | 4.15 | 4.70 | 5.39 | 6.03 | 6.95 | 9.30 | 13.06 | 16.91 | 26.07 |
|  | Sen%/spe% | 59.4/61.5 | 53.3/67.9 | 50.6/75.3 | 45.5/80.1 | 37.2/80.1 | 33.0/84.1 | 24.5/85.1 | 20.8/93.2 | 13.1/93.2 | 6.3/94.3 |
|  | MetS (%) | 72.9 | 74.0 | 78.1 | 79.9 | 76.5 | 78.3 | 74.2 | 84.3 | 77.1 | 65.7 |
| **Age** |  |  |  |  |  |  |  |  |  |  |  |
| 25-34 yr | Cut | 3.20 | 3.58 | 3.95 | 5.42 | 5.69 | 7.43 | 8.01 | 9.31 | 15.90 | 18.0 |
|  | Sen%/spe% | 78.4/32.8 | 78.4/51.0 | 78.4/72.6 | 66.6/72.6 | 52.3/78.0 | 40.5/83.4 | 40.5/83.4 | 40.5/94.6 | 28.8/94.6 | 28.8/100 |
|  | MetS (%) | 35.0 | 42.4 | 56.9 | 52.8 | 52.3 | 53.0 | 53.0 | 77.5 | 71.0 | 100 |
| 35-44 yr | Cut | 4.32 | 4.41 | 5.21 | 5.34 | 6.95 | 7.50 | 9.44 | 14.86 | 23.46 | 26.54 |
|  | Sen%/spe% | 61.4/56.4 | 58.4/60.5 | 50.3/69.2 | 47.3/73.9 | 40.7/80.2 | 30.7/80.2 | 28.2/86.5 | 21.7/90.6 | 12.4/90.6 | 8.8/95.3 |
|  | MetS (%) | 65.3 | 66.4 | 68.6 | 70.8 | 73.4 | 67.5 | 73.7 | 75.5 | 63.7 | 71.5 |
| 45-54 yr | Cut | 3.33 | 3.53 | 4.15 | 4.67 | 4.89 | 5.25 | 5.77 | 6.67 | 8.10 | 11.97 |
|  | Sen%/spe% | 54.9/58.9 | 49.1/62.4 | 45.0/71.5 | 40.4/78.5/ | 33.2/78.5 | 30.8/88.4 | 24.7/88.4 | 17.4/91.5 | 11.4/94.0 | 5.5/94.0 |
|  | MetS (%) | 77.7 | 77.3 | 80.5 | 83.0 | 80.1 | 87.3 | 84.7 | 84.2 | 83.3 | 70.6 |
| 55-64 yr | Cut | 3.17 | 3.36 | 3.58 | 3.91 | 4.50 | 5.17 | 6.03 | 6.89 | 8.79 | 14.93 |
|  | Sen%/spe% | 49.5/47.6 | 45.1/55.0 | 39.6/57.4 | 35.9/64.9 | 31.4/72.4 | 25.9/74.8 | 19.7/77.3 | 16.1/89.9 | 12.6/9.8 | 5.4/94.8 |
|  | MetS (%) | 72.3 | 73.4 | 72 | 73.8 | 75.9 | 74.0 | 70.5 | 81.4 | 87.1 | 74.3 |

^a^ weighted and standardized for age and sex, ^b^ for age, and ^c^ for sex distribution of the population of Iran in 2006.

^1^: IDF definition

**Table S4.** Summary of reports (sorted by sample size) on HOMA-IR cut-off in different populations

| **Ref.** | **Characteristics of study subjects** | **Threshold value** |
| --- | --- | --- |
| [6] | 4,816 Sweden, population-based sample | ≥ 2.0 (75^th^ percentile) |
| [7] | 2,264 Brazilian, age: 4 – 93 yr | <18 yr: 2.39 ± 1.93  ≥ 18 yr: 3.20 ± 3.56 |
| [8] | 1,898 Brazilian, age: 18 – 90 yr with normal fasting glucose | Total: 1.8 ± 0.9  BMI: < 25: 1.2 ± 0.65  25 – 30: 1.8 ± 0.98  >30: 2.9 – 1.6 |
| [9] | 1317 Brazilian, age: 40 ± 12 yr, BMI: 34 ± 10 kg/m^2^ | ≥ 2.77 (90^th^ percentile) |
| [10] | 1,276 Iranian, age: 38 ± 12 yr, non-diabetic, normotensive | IDF-MetS: ≥1.80 (ROC)  ATPIII-MetS: ≥1.95 (ROC)  ≥ 1.6 (75^th^ percentile)  ≥ 1.8 (80^th^ percentile)  ≥ 2.3 (90^th^ percentile) |
| [11] | 976 Korean, age: 30 – 79 yr, non-diabetic | ≥ 2.34 (ROC) |
| [12] | 490 Spanish, age: 19 - 70 yr, BMI: 26.3 ± 4.4 kg/m^2^, non-diabetic | 2.7 ± 0.1 |
| [13] | 225 Italian, age: 40 - 79 yr, , healthy subjects with no metabolic disorders | ≥ 2.77 (80^th^ percentile) |
| [14] | 161 Japanese, age: 41.6 ± 0.4 yr, healthy subjects with no metabolic disorders | ≥ 1.7 (90^th^ percentile) |
| [15] | 140 Spanish, age: 7 - 16 yr | approximately 3 (ROC) |
| [16] | 120 Chinese, age: 19 - 40 yr, with normal BMI and with normal fasting glucose | 1.96 ± 0.57 |
| [17] | 97 Spanish, age: 20 - 65 yr, BMI: 22.2 ± 1.9 kg/m^2^ | ≥ 3.8 (90^th^ percentile) |
| [18] | 57 Turkish, obese adolescents | ≥ 3.16 (ROC) |
